# Buffalo Hospital of the Sisters of Charity

**Published:** 1849-09

**Authors:** F. H. Hamilton

**Affiliations:** Attending Surgeon, &c.


ART. IV.—*Buffalo Hospital of the Sisters of Charity. Surgical Department. Selected Cases: by* F. H. HAMILTON, A. M., M. D., Attending Surgeon, &c.

*Case* 1. *Dislocated Shoulder;—Chloroform.* Mrs. Mary Ann Hasler, aet. 47 years; admitted Dec. 20, 1848. Nearly three weeks since, she fell, striking on the radial side of the humerus, about three inches below its head, and dislocating the humerus into the axilla. An empiric saw it within fifteen minutes; it was not then swollen, but he said it was not dislocated. On the fifth or sixth day following, a Catholic clergyman attempted to reduce it.

When admitted, the patient was examined by Drs. Flint, McCall, and Gilfillen, as well as by myself. The arm was lengthened one quarter of an inch; no swelling existed, and the head of the humerus could be distinctly felt in the axilla.

The patient was laid upon a mattress, and two drachms of chloroform administered by inhalation. When a partial influence was obtained, I placed my heel in the axilla, and made extension upon the arm. In about thirty seconds the bone slid into its place.

This woman had been a long time subject to a harrassing cough, yet the inhalation of the chloroform did not incommode her.

*Case* 2. *Pterygeum*; *Removal.* Mrs. P——, aged about 40 years: has had pterygeum in both eyes twenty years. On the right eye the pterygeum is fleshy, internal, and reaches quite to the centre of the cornea. The patient was submitted to absolute diet during four days, and then we operated upon the right eye, by raising that portion of the pterygeum which covered the sclerotica, with a pair of toothed forceps, and, with the scissors, excising freely about half an inch of its length; terminating the excision at the border of the cornea. The entire breadth of the enlarged structure was removed, also, and about one line of the conjunctiva beyond its borders.

No applications were directed except tepid water. The cure was radical in three weeks.

*Case* 3. *Staphyloma racemosa.* Patrick Dugan, aet. eight months; admitted Nov. 7, 1848. About five weeks previously the eye had become inflamed; had no medical attendance for two weeks. Ulceration of the cornea and evacuation of the anterior chamber, had then occurred.

When this child was admitted the cornea was thin and bulging irregularly, and completely opaque.

The treatment consisted in repeated evacuations of the anterior chamber, through small openings in the cornea made by an iris knife, and in the application of a solution of nitras argenti, grains four to an ounce of water, once daily. The cornea was opened eight times, and after each operation the corneal projection diminished sensibly. The result, after a few weeks, has been a restoration of the natural form of the eye, and the removal of much of the opacity with a re-establishment of tolerable vision: a result not often obtained.

We regard the application of the nitras argenti as a valuable auxiliary in the treatment. It not only arrests the diseased action upon which the staphyloma subsists, and removes the corneal opacity, but it probably contracts and hardens the cornea, and thus diminishes the protrusion.

*Case* 4. *Tonsillotomy; Profuse Hæmorrhage.* Benjamin Reiser; aet. 22 years. His tonsils began to enlarge about three years since, and are now much larger than the average of those which we are called upon to excise. A few weeks before the operation, Nov. 30, '48, he had a severe attack of bronchitis.

The size of the tonsils, and the late period of life at which they began to enlarge, are unusual, and, with the tendency to bronchitis, indicate, we think, a strong tubercular inclination. A suspicion that the diseased tonsils might have had some agency in the production of the bronchitis, was the pricipal apology for excising them.

We removed one of the tonsils in presence of the class, in the usual manner, with Owen's tonsillotome. Three-fourths of the gland was brought away. A slow but steady hæmorrhage followed, which was not arrested short of slight syncope. The soreness of the throat which followed was also much more severe and protracted than usual.

Although urged very strongly, we have since declined operating upon the other tonsil, from a fear of both the immediate and remote consequences of such copious bleedings: consequences which are especially to be dreaded if he actually labors under a tubercular diathesis.

*Case* 5. *Burn.* Admitted Nov. 22, 1848. Mary J. Laudwehr, aet. 24. Nov. 19, while working near the kitchen fire, over which hung a kettle of fat, the fat took fire, and the blaze communicating to her clothes, burned her face, neck, both arms and hands, with a portion of her thorax. The burn was both superficial and ulcerated, and was attended with great pain and high febrile action. During the five first days, the burns were dressed with carded cotton and sweet oil. On the 24th the lime water liniment and linen cloths were substituted. Dec. 3 she was attacked with diarrhœa and vomiting, and her pharynx became very much inflamed; circumstances which seemed to be connected with the great irritation existing upon the skin.

Dec. 9, discharged cured.

*Case* 6. *Burn.* Harriet O'Neil, aet. 19 years, admitted July 14, 1848; arms, hands, chest, and both legs extensively and deeply burned by her clothes having taken fire. At first carded cotton and sweet oil were applied; subsequently, the lime water dressings were used, and under this latter treatment the ulcers rapidly cicatrized.

Dismissed Aug. 4, 1849.

In neither of the above cases have contractions of the integuments, tendons, or fasciæ taken place.

*Case* 7. *Amputation at metacarpo-Phalangeal Articulation—chloroform.* Stephen Clifford, aet. 25, admitted Sept. 5, 1848. In consequence of a fracture which occurred five weeks before, a sloughing of the soft parts of the finger had occurred.

Having rendered the patient partially insensible by inhalation of chloroform, we made the amputation (fore-finger) at the Metacarpo-phalangral articulation.

*Process.* A single ovoid flap was made from the radial side of the finger, and the joint opened, and the disarticulation effected from the same direction. No sutures or ligatures were employed. The wound was closed with adhesive straps. Union occurred in the usual time.

*Case* 8. *Lumbar Abscess; Chronic; Accompanied with Caries of the Spine.* Mrs. Mary Murphy, aet. 26 years; admitted Nov. 10, 1848. Mrs. M. felt a pain in the lumbar region six months before admission.

Three days before admission an abscess opened just below the anterior, superior spinous process of the ileum, and above Poupart's ligament. The discharge was serous, with a few flakes of lymph. The general health of the patient was good, and she knew of no cause for the disease.

Jan. 25, 1849, she had her first chill. The chills, with fever and night sweats, occurred occasionally thereafter as long as she was in the Hospital.

March 12, 1849, her lumbar vertebræ were found to have become carious, and a lateral curve had occurred.

May 31 she left the Hospital to return to the house of a friend. The disease was still progressing.

Mrs. M. was treated locally by counter irritants, in the early history of the case; and, constitutionally, by such remedies as iodide of potash, iron, quinine, mineral acids, mineral waters, wine, good diet, &c. throughout most of the time. At first she was allowed exercise, but, after the spine became curved, rest in the recumbent posture was generally observed.

The points especially worthy of notice are the extremely slow progress of the caries; the existence of a considerable abscess without chills; the spontaneous evacuation of the abscess and free admission of the air without constitutional irritation; the condition of almost uninterrupted good health until the chills did occur, which was full nine months after the inception of the disease.

We believe that under judicious treatment she will yet recover.

